# Biliary Dilatation While Awaiting Surgery for a Congenital Hiatal Hernia: A Case Report

**DOI:** 10.1111/ases.70212

**Published:** 2025-12-17

**Authors:** Ryuta Masuya, Jun Kuwabara, Katsuya Watanabe, Satoshi Ieiri, Taro Oshikiri

**Affiliations:** ^1^ Department of Gastrointestinal Surgery and Surgical Oncology Ehime University School of Medicine Toon City Ehime Japan; ^2^ Department of Pediatric Surgery, Research Field in Medical and Health Sciences, Medical and Dental Area, Research and Education Assembly Kagoshima University Kagoshima Kagoshima Japan

**Keywords:** biliary dilatation, cholestasis, congenital hiatal hernia, laparoscopic surgery

## Abstract

Giant congenital hiatal hernias that cause biliary dilatation are uncommon. We present the case of a female neonate with a massive hiatal hernia involving the entire stomach, which was located in the mediastinum, who developed cholestasis presenting with elevated bilirubin and grayish stools, along with dilatation of the intrahepatic and common hepatic ducts by 90 days of age. The common bile duct remained undilated and no pancreatic herniation was evident. A laparoscopic hernia repair was performed at 141 days. Intraoperative cholangiography suggested that hernia‐induced common bile duct kinking caused the stasis; consequently, no biliary surgery was performed. Postoperatively, the liver function and bilirubin levels normalized, although MRI at 2 months revealed residual ductal dilatation. Neonatal hiatal hernias can induce biliary dilatation through mechanical kinking, even without pancreatic prolapse. While hernia repair may resolve cholestasis, persistent ductal alterations require long‐term monitoring.

## 1 Introduction

Giant hiatal hernias, in which the entire stomach protrudes into the mediastinum, are primarily reported in adults, although congenital cases with immediate postnatal symptoms also occur. Cholestasis associated with giant hiatal hernias has been documented in only a few adult cases and no previously reported pediatric cases. This report presents a case of congenital hiatal hernia that developed biliary dilatation due to cholestasis, necessitating differentiation from congenital biliary dilatation.

## 2 Case Presentation

**FIGURE 1:**
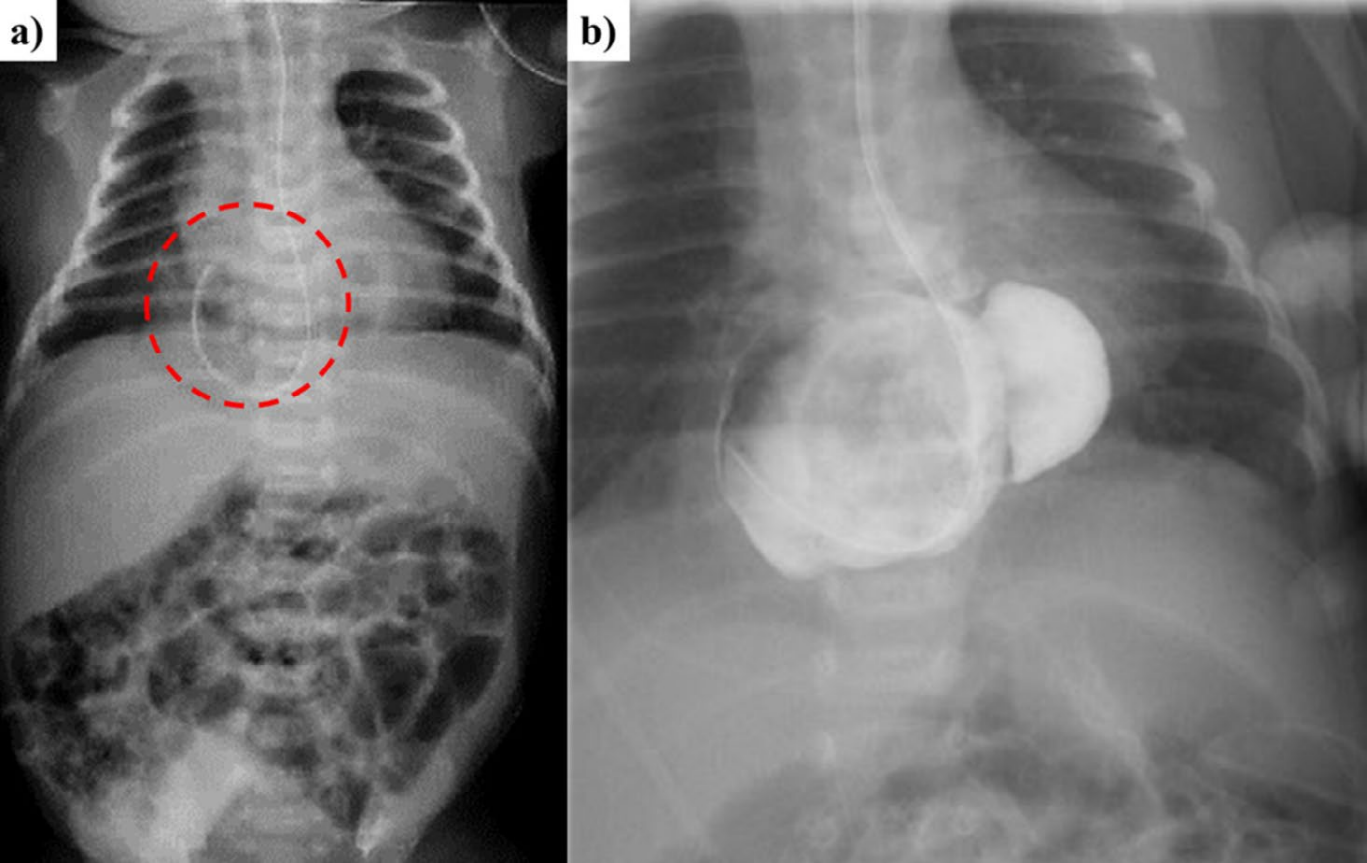
Preoperative radiography findings. (a) Plain radiography shows a gastric tube coiled up at the diaphragm level (red circle). (b) Contrast radiography showed a hiatal hernia with the entire stomach prolapsing into the mediastinum.

A female infant (birth weight, 2712 g; Apgar, 8/9) was delivered via planned cesarean section at 38 + 2 weeks due to a maternal history of myomectomy. The pregnancy was uneventful. Immediately after birth, the neonate exhibited retraction and tachypnea, requiring NICU admission for impaired lung aeration. Admission X‐rays showed that the gastric tube was coiled at the diaphragm level (Figure 1a). Upper gastrointestinal contrast studies revealed a hiatal hernia, with the entire stomach protruding into the mediastinum (Figure 1b). Surgery was planned after weight gain was achieved through continuous gastric tube feeding.

**FIGURE 2:**
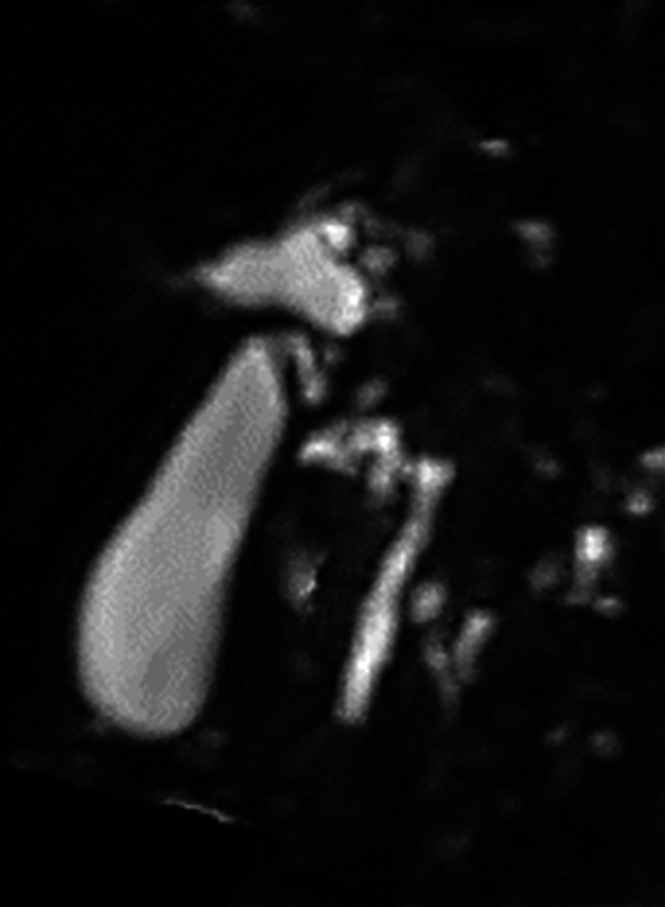
Preoperative magnetic resonance imaging (MRI) findings. MRI findings at 126 days of age revealed dilatation of the intrahepatic bile ducts, common hepatic duct, and gallbladder. The common hepatic duct ran to the left side. Dilatation of the common bile duct was not observed. It was difficult to identify the presence or absence of a pancreaticobiliary maljunction.

At approximately 30 days of age, transient grayish‐white stools and elevated direct bilirubin levels were observed. After 90 days, serum liver enzyme levels and direct bilirubin levels increased. Magnetic resonance imaging (MRI) at 126 days (Figure 2) showed dilatation of the intrahepatic bile ducts, common hepatic duct, and gallbladder; the common bile duct was not dilated. Pancreatic extrusion into the mediastinum was not observed (Figure S1). Preoperative evaluation of the pancreaticobiliary maljunction was difficult.

Laparoscopic repair was performed under general anesthesia at 141 days of age (Video S1). At the time of surgery, the height was 58 cm (−2.08 SD) and the weight was 5.54 kg (−1.24 SD). The herniated stomach was repositioned into the abdominal cavity without difficulty because no adhesions were present. The hernia sac, formed by the elongated phrenoesophageal ligament, was incised and the abdominal esophagus was taped. The esophageal hiatus was repaired with sutures, and the His angle was recreated by suturing the fundus, abdominal esophagus, and the left diaphragmatic crus. Anterior gastropexy was performed.

**FIGURE 3:**
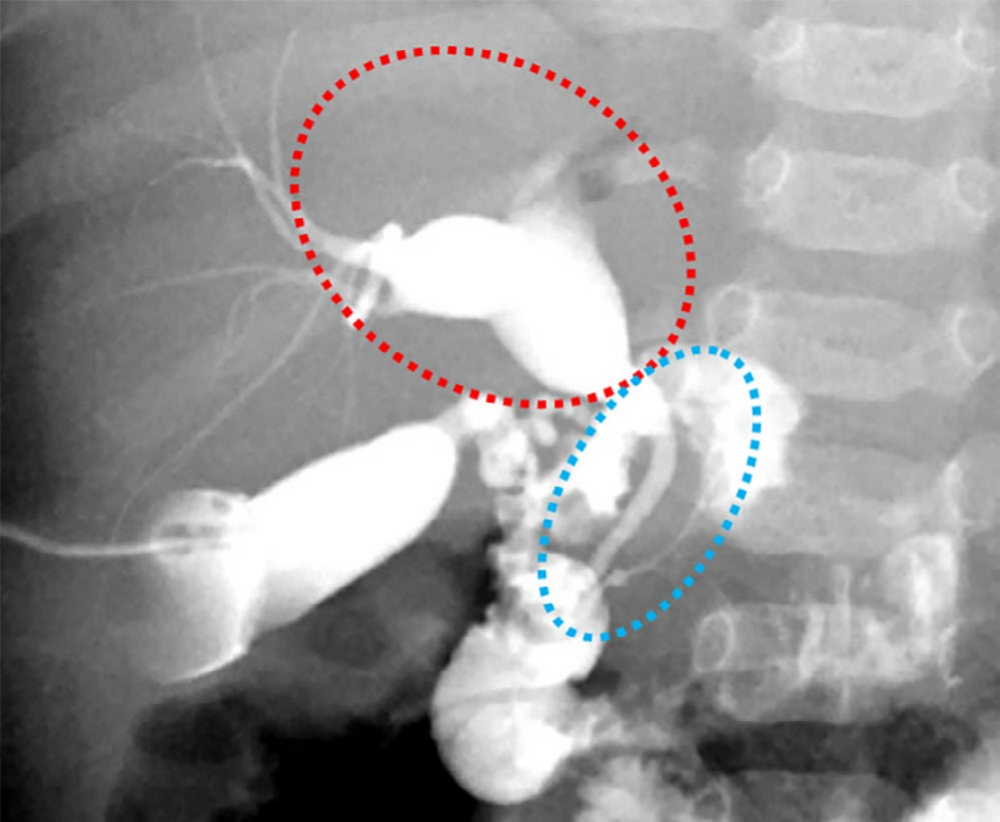
Intraoperative cholangiography after the replacement of the stomach. Intraoperative cholangiography showing dilatation of the intrahepatic bile ducts and the common hepatic duct (red circle). The common bile duct is not dilated (blue circles). There was not a pancreaticobiliary maljunction.

Intraoperative cholangiography (Figure 3), performed after hernia repair, revealed dilated intrahepatic and common hepatic ducts. The common hepatic duct ran toward the left side. The common bile duct was not dilated, and pancreaticobiliary maljunction was not observed. Gallbladder bile biochemistry revealed pancreatic enzyme levels equivalent to serum levels, ruling out significant retrograde pancreatic fluid flow. We concluded that bile duct dilatation resulted from kinking in the common bile duct caused by the hiatal hernia and thus opted against hepatic duct resection or hepaticojejunostomy.

Postoperatively, the patient showed no gastroesophageal reflux symptoms, tolerated oral intake, and gained weight. Liver enzyme levels showed transient elevation but normalized without recurrence, and bilirubin levels remained normal. MRI at 9 months post‐surgery showed residual dilation of the intrahepatic and common hepatic ducts (Figure S2).

## 3 Discussion

Congenital hiatal hernia, especially in massive forms in neonates causing respiratory and feeding issues, is rare [1, 2]. Our patient presented with neonatal respiratory distress and was diagnosed with a massive hiatal hernia (the entire stomach in the mediastinum) via imaging, which was confirmed by upper gastrointestinal contrast studies.

Our patient developed cholestasis (grayish‐white stools, elevated direct bilirubin, and liver enzymes) approximately 1 month postnatally, with imaging showing dilatation of the intrahepatic and common hepatic ducts, but not the common bile duct. While biliary complications with hiatal hernias are rare, adult reports often link them to pancreatic herniation, causing common bile duct compression or kinking [3, 4, 5]. Such herniation of the pancreas or duodenum can lead to obstructive jaundice [4, 5]. For instance, Miyagishima et al. described bile duct stenosis with pancreatic herniation [3] and Furtado et al. reported surgical relief of common bile duct obstruction due to pancreatic herniation with axial displacement [4]. Whereas several adult cases of hiatal hernia causing biliary obstruction have been reported, a PubMed search suggests that no pediatric cases have been described.

In contrast, our patient had no pancreatic herniation. However, intraoperative cholangiography revealed an abnormally leftward‐deviated common hepatic duct, likely due to the hernia, causing kinking, stasis, and subsequent dilatation. This suggests that biliary compromise can occur from hiatal hernias via traction or displacement of the adjacent structures, even without direct pancreatic compression.

Differentiating this condition from congenital biliary dilatation and biliary atresia is crucial. Intraoperative cholangiography revealed no pancreaticobiliary maljunction, and normal biliary pancreatic enzyme levels excluded significant reflux, making congenital biliary dilatation (especially with maljunction) unlikely. The absence of common bile duct dilatation has also been argued against typical congenital biliary dilatation.

Postoperatively, the liver function and bilirubin normalized rapidly, suggesting that hernia repair corrected the common bile duct bend and relieved the stasis. However, MRI at 2 months showed residual intrahepatic and common hepatic duct dilatation. While such dilatation may resolve slowly or persist, several reports have demonstrated favorable long‐term surgical outcomes in pediatric giant hiatal hernias with limited detail on biliary issues [6, 7]; therefore, persistent dilatation warrants caution. The risk of recurrent liver dysfunction or future cholelithiasis necessitates careful long‐term follow‐up with regular imaging and liver function testing.

In conclusion, this rare case of neonatal giant hiatal hernia with biliary dilatation suggests mechanical kinking of the common bile duct even in the absence of pancreatic herniation. The patient's liver function improved post‐repair, but persistent ductal dilatation mandated strict long‐term follow‐up. This case highlights this rare complication and its management.

## Author Contributions

All authors were involved in the patient treatment, ideation, manuscript writing, and critical review. Specific authors carried a more significant responsibility: as the principal investigator, Ryuta Masuya takes responsibility for all the work done on the manuscript. Ryuta Masuya, Dr. Jun Kuwabara, and Dr. Katsuya Watanabe performed surgery and managed the postoperative state. Ryuta Masuya drafted the manuscript. Dr. Jun Kuwabara, Dr. Katsuya Watanabe, Prof. Satoshi Ieiri, and Prof. Taro Oshikiri critically revised the manuscript for crucial intellectual content.

## Ethics Statement

All procedures performed in this study were reviewed by our Institutional Research Committee, and the study was performed in accordance with the ethical standards of the 1964 Declaration of Helsinki and its later amendments. We obtained consent from the patient's family for the publication of this article.

## Conflicts of Interest

Dr. Satoshi Ieiri is an Editorial Board member of ASES Journal and a co‐author of this article. To minimize bias from all editorial decision‐making related to the acceptance of this article for publication. The other authors declare no conflicts of interest in association with the present study.

## Supporting information


**Figure S1:** Preoperative magnetic resonance imaging (MRI) findings. The pancreas was not herniated (arrowhead).

**Figure S2:** Postoperative magnetic resonance imaging (MRI) findings. MRI at 9 months post‐surgery showed residual dilatation of the intrahepatic duct.

**Video S1:** Intraoperative findings. The herniated stomach was easily repositioned. The hernia sac was incised and the abdominal esophagus was taped. The esophageal hiatus was repaired with sutures, and the His angle was recreated by suturing the fundus, abdominal esophagus, and the left diaphragmatic crus. Anterior gastropexy was performed.

## Data Availability

Data sharing is not applicable to this article, as no datasets were generated or analyzed during the current study.
