# Supplementary figures and images for: Biliary Dilatation While Awaiting Surgery for a Congenital Hiatal Hernia: A Case Report
Source: Asian J Endosc Surg. 2025 Dec 17;18(1):e70212. doi: 10.1111/ases.70212 (PMC12711113; doi:10.1111/ases.70212)

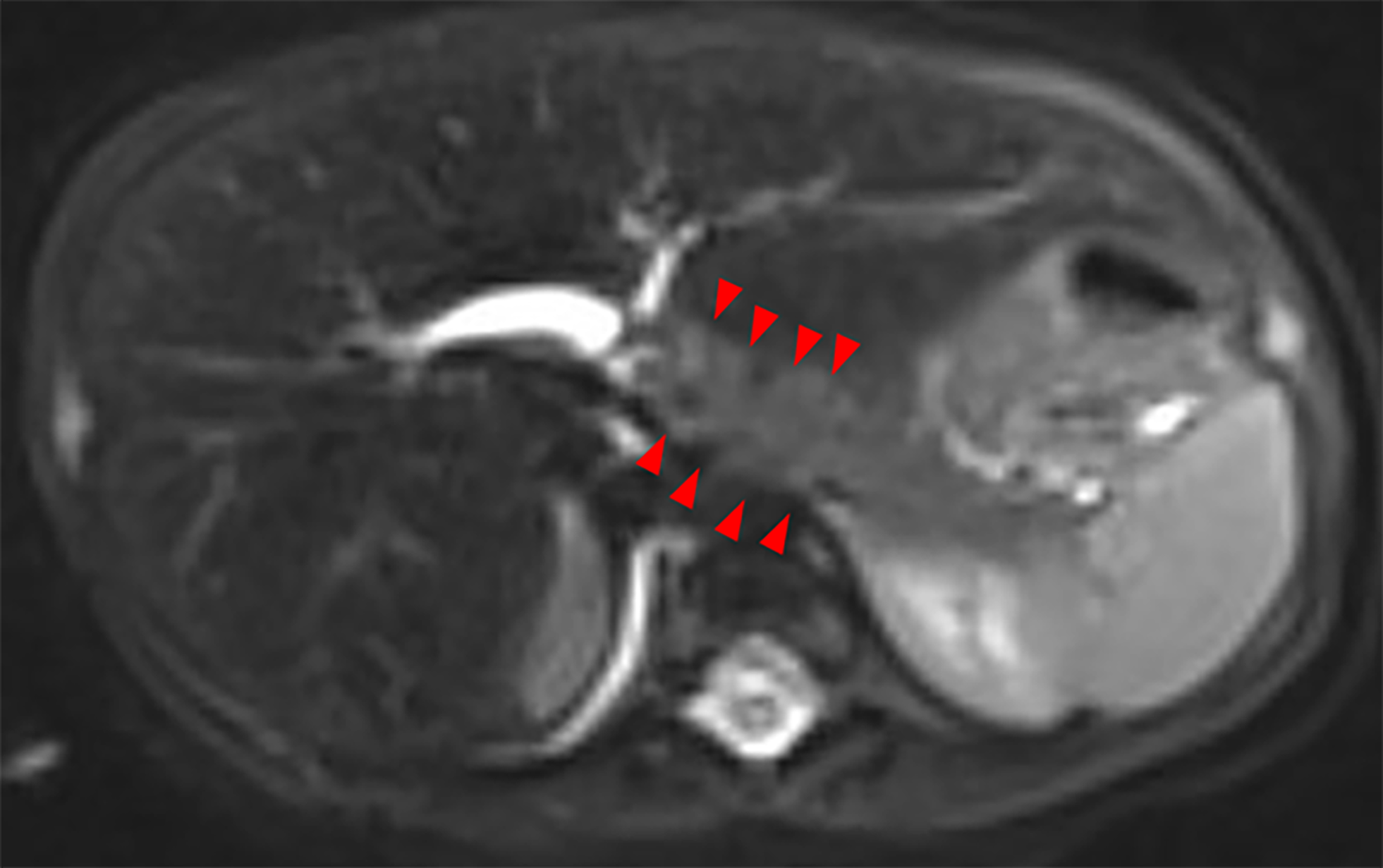

Supplement: Supplementary file 1 — Figure S1: Preoperative magnetic resonance imaging (MRI) findings. The pancreas was not herniated (arrowhead). [file ASES-18-e70212-s003.tif]

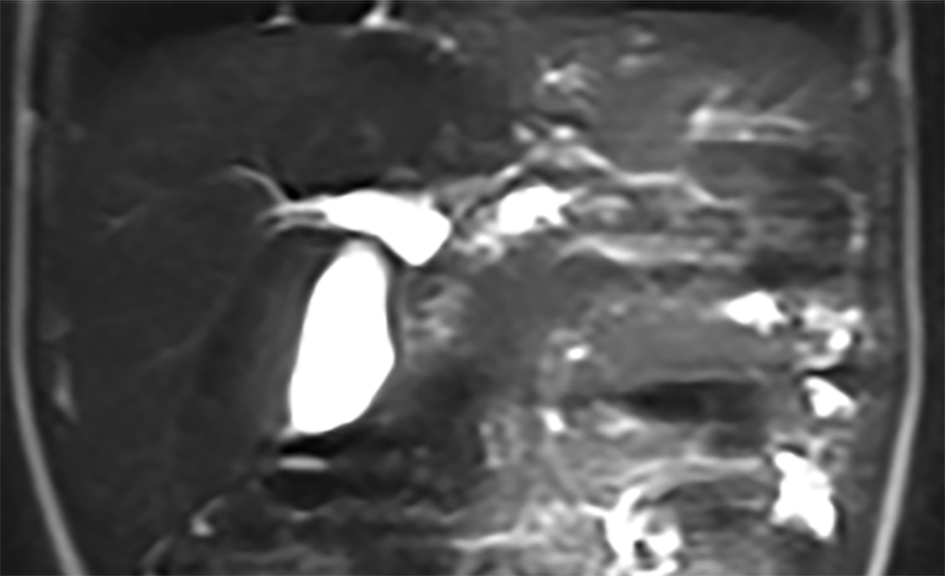

Supplement: Supplementary file 2 — Figure S2: Postoperative magnetic resonance imaging (MRI) findings. MRI at 9 months post‐surgery showed residual dilatation of the intrahepatic duct. [file ASES-18-e70212-s002.tif]
